# Algorithms for Smooth, Safe and Quick Routing on Sensor-Equipped Grid Networks

**DOI:** 10.3390/s21248188

**Published:** 2021-12-08

**Authors:** Giovanni Andreatta, Carla De Francesco, Luigi De Giovanni

**Affiliations:** Dipartimento di Matematica “Tullio Levi-Civita”, Università degli Studi di Padova, 35122 Padova, Italy; giovanni.andreatta@unipd.it

**Keywords:** automated transportation network, collision-free routing, grid network, optimization algorithm, integer linear programming, heuristics

## Abstract

Automation plays an important role in modern transportation and handling systems, e.g., to control the routes of aircraft and ground service equipment in airport aprons, automated guided vehicles in port terminals or in public transportation, handling robots in automated factories, drones in warehouse picking operations, etc. Information technology provides hardware and software (e.g., collision detection sensors, routing and collision avoidance logic) that contribute to safe and efficient operations, with relevant social benefits in terms of improved system performance and reduced accident rates. In this context, we address the design of efficient collision-free routes in a minimum-size routing network. We consider a grid and a set of vehicles, each moving from the bottom of the origin column to the top of the destination column. Smooth nonstop paths are required, without collisions nor deviations from shortest paths, and we investigate the minimum number of horizontal lanes allowing for such routing. The problem is known as fleet quickest routing problem on grids. We propose a mathematical formulation solved, for small instances, through standard solvers. For larger instances, we devise heuristics that, based on known combinatorial properties, define priorities, and design collision-free routes. Experiments on random instances show that our algorithms are able to quickly provide good quality solutions.

## 1. Introduction

Modern transportation and handling systems greatly benefit from information technology (IT) and automation, as demonstrated by the consolidated use of sensor-equipped transport networks, automated guided vehicles (AGVs), self-moving robots, as well as the growing adoption of drones, in many industrial, logistic and public transportation environments. Typical examples can be found in railway transportation systems, where optic or acoustic sensors on trains and tracks, integrated by collision detection and avoidance logic, support safe and efficient operations. IT also supports taxiways operations in airports airside [1], where aircraft, passenger buses as well as many ground service vehicles (like baggage dollies, passenger steps, tow-tractors, follow-me cars, etc.) run intersecting routes between the boarding gates and the runways, and the risk of collisions or deadlocks has to be constantly monitored. Another application in logistic networks involves the use of AGVs in port terminals [2] to transport containers from the berths on the quay along the shoreline to dockside stacks and land access points. In a similar way, automated warehouses or factories adopt vehicles (like AGVs or drones) to transfer goods or materials from the depot shelves to the delivery docks or between production lines [3], or to perform other inventory, inspection or surveillance operations [4,5]. In all of these cases, the traffic load may be relevant and appropriate vehicle routes must be designed and operated, in order to mitigate the risk of collision while preserving the system efficiency in terms of transportation time and cost. To this end, IT provides hardware and software devices to support safe and efficient transport network operations. A sensing network, including sensors installed on the network infrastructure and/or the vehicles, collects information (position, direction, velocity, etc.), that is processed by software logic that schedules vehicle movements and detects possible conflicts or deadlocks. In this context, the availability of optimization algorithms can be determinant in reducing transportation time, cost and accident rates, with relevant economic as well as social benefits.

We focus on automated transportation systems where, for the sake of safety, potential collisions should be avoided in advance as much as possible. In particular, we envision a system where, given the initial and the goal positions of each vehicle, a set of collision-free *nominal* routes are determined, and the sensing network and related logic manage, at real-time, possible conflicts or deadlocks that may arise due to unpredicted events causing any vehicle or network disruptions that prevent following the predefined schedule. Moreover, in many cases, like, container port terminals (see, e.g., [6]), logistic and industrial warehouses, etc., automated transportation systems rely on a grid network topology, where the vehicle moves on intersecting horizontal and vertical lanes. This motivates us to consider a simplified, although realistic, setting, where vehicles are initially positioned on one side of the grid network (e.g., the berths along the shoreline in a port terminal, or the gates of an airport apron) and have to reach a destination on the opposite side (e.g., the land or the runways access points), and the time needed to move between any two consecutive lanes is the same for all vehicles. Under these settings, the most efficient way for a single vehicle to reach its destination is to follow a smooth *nonstop* path that starts from the origin and only contains moves on horizontal and vertical lanes in the same direction, the one towards the destination. Such kinds of shortest paths on the grid do not contain horizontal (or vertical) moves in opposite directions and are called *Manhattan paths*. Clearly, there exists more than one Manhattan path for each vehicle. If a fleet of two or more vehicles has to be routed, choosing Manhattan paths may cause collisions, since two vehicles may require to cross the same intersection or the same road segment between two lines at the same time. Collisions can be avoided by choosing different Manhattan paths rather than stopping vehicles along the path, in order to preserve efficiency. To this end, let us consider, without loss of generality, the case where vehicles have to move from the bottom side of the grid to the top side and observe that it is always possible to route vehicles, without stops, on a set of collision-free Manhattan paths where each vehicle performs all the required horizontal moves on a different dedicated horizontal lane. The drawback of such a solution is the possibly large number of required horizontal lanes, which corresponds to long displacement times and large infrastructural and operational costs, including, e.g., land consumption, sensing network installation and operation and transportation costs. On the other hand, a small number of available horizontal lanes may not be sufficient to guarantee the possibility of finding collision-free routes without stopping or deviating from the Manhattan paths.

The question of determining the smallest number of required horizontal lanes is the object of the fleet quickest routing problem on grids (FQRP-G), which can be stated as follows. We are given a grid network made of intersecting horizontal and vertical lanes and a set of vehicles. The time to move between consecutive lanes is constant and the same for every vehicle. Each vehicle is initially positioned at its origin at one side of the grid and has to reach its destination at the opposite side: without loss of generality, let origins be located at the bottom and destinations at the top of the grid, that is, the route of each vehicle starts at the bottom of a vertical lane and ends at the top of a (possibly different) vertical lane. We want to determine the minimum number of horizontal lanes that allow routing the vehicles on a set of collision-free nonstop Manhattan paths.

The scope of the paper is presenting an exact solution approach to FQRP-G, based on mathematical programming, and alternative fast heuristics that exploit relevant theoretical results presented in the literature, with the aim of assessing their computational performance and their impact on the design of time-cost efficient and safe routing systems. After reviewing the literature related to FQRP-G in Section 2, the general methodology adopted in this paper, based on modelling the problem on an undirected grid graph, is presented in Section 3, together with previous theoretical results that are relevant for our work, and an integer linear programming formulation of FQRP-G. Fast heuristic algorithms are reported in Section 4, one corresponding to a more efficient implementation than that proposed in [7], and further greedy procedures that prioritize vehicles based on measures computed on a conflict graph, defined in Section 3.2. Section 5 reports on computational experiments on a benchmark of more than 200 random and on-purpose designed instances of different sizes up to 300 columns and vehicles. Results show that the proposed exact approach is able to solve instances up to about 150 vehicles in a few seconds, whereas running times become longer than one minute, and exponentially increase for larger instances. In any case, we show that the optimal solution, on average, would enable large per cent savings in terms of required horizontal levels. The tested heuristics always run in a blink. Moreover, even if the gap from the optimal solution may be, in theory, very large, the worst-case performance is just observed on on-purpose designed instances, whereas the performance on random instances, in particular for the first heuristic, shows just a few additional required horizontal lanes with respect to the optimal solution. This means that, as discussed in Section 6, the proposed heuristic can be used in realistic settings with relevant savings in terms of transportation and sensing infrastructure while preserving vehicle route safety. The concluding Section 7 summarizes the findings of the paper and draws some lines for further research.

## 2. Literature Review

Several works in literature are related to FQRP-G and, more generally, to designing a routing network and finding collision-free schedules for multiple vehicles.

In the collision-free route planning problem (also known as *multi-agent path finding* in artificial intelligence literature), a set of vehicles with a given origin and destination has to move in a given routing network, modelled as a directed graph. A first group of papers presents *static* approaches, where, for each vehicle, nominal routes are computed on the underlying routing network, taking load-balance factors into account, to prevent collisions as far as possible [8,9], or, if the application context allows, by dividing the routing area into non-intersecting zones, each occupied by one vehicle at a time, as in regional control models [9,10,11,12]. In general, such approaches cannot guarantee collision-free nominal routes, and additional methods are required during their execution to detect and resolve collisions and, in case, deadlocks, based on, e.g., Petri Net approaches [13,14], graph-theoretic models [15], queries on geospatial reference grid systems [16] and searching the space of possible deviations from nominal routes [17]. A specialized static approach for grid networks is presented in [18], where initial routes that minimize collisions are chosen from equivalent Manhattan paths, and selected collision avoidance rules, based on preliminary collisions classification, are applied during execution.

An improved static method is proposed in [19], where statically computed load-balanced paths are post-processed by resource reservation and deadlock prevention techniques inspired by [20], leading to collision-free routes.

Notice that, in general, collision and deadlock avoidance introduce deviations from nominal shortest paths as well as delays in the vehicle schedule, since stops may be required during routes operation. By considering deviations and delays already at the planning stage, *dynamic* approaches are able to directly determine optimized collision-free paths and schedules, by taking into account that the impact of vehicle routes on network resources changes over time. For a general network topology, the authors of [21] developed a heuristic based on a mathematical programming formulation and column generation, whereas exact algorithms are devised in [22] for the special case where the routing network consists of two horizontal lanes and vertical bridges between them, and in [23] for the special case of two vehicles on a grid network. The dynamic approach proposed in [24] for the general case, iteratively computes shortest paths on a time-expanded network, and it is suitable for online settings, where transportation requests may appear during operations. In [25], a time-expanded network allows dynamically modelling the problem as a multi-commodity network flow [26]: the corresponding integer linear programming formulation, solved by state-of-the-art off-the-shelf solvers, provides either cost- or time-optimal schedules for up to 50 vehicles to be routed on a grid network.

The literature also integrates collision avoidance methods and dynamic algorithms with general heuristic searching techniques (A*-based search, evolutionary algorithms, particle swarm optimization, neighbourhood search, etc. [27]) that efficiently explore different vehicle priorities and conflict-resolution policies, as well as alternative routes towards the destinations. For example, in [28], an improved A* algorithm searches the paths between vehicles origin and destination in a grid network related to a warehouse environment, also taking congestion measures into account, and grid specific priority rules are used to solve residual conflicts. In [29], the D* Lite search algorithm is run on a reachability graph obtained from a suitable coloured Petri net that models feasible multi-AGV trajectories. A Time Enhanced A* search is proposed in [30] to find collision-free route plans in a time-expanded network, and integrated with tabu search techniques to further improve the efficiency by changing the assignment of transport tasks between robots. The Conflict Based Search proposed in [31], and further enhanced in [32], explores a constraint tree whose nodes are evaluated through nominal shortest routes and, in case of collisions, branches are generated corresponding to alternative vehicle priorities. For the solution of a real ship traffic optimization problem, the authors of [33] integrate the dynamic collision-free routing algorithm proposed in [24] into a local search scheme that explores the space of possible alternative scheduling decisions related to precedence conflicts between ships that compete for traversing a waterway with limited capacity and equipped with sidings to allow ships stopping and passing each other, according to the chosen precedence strategy.

We remark that the collision-free routing methodologies described above allow for vehicle stops and deviations from the nominal shortest paths (Manhattan paths, in the case of grids), whereas our research focuses on smooth nonstop routes. With this respect, FQRP-G has relations to the design of at-grade traffic networks without conflicts, aiming at configuring and operating a routing network where all roads run at the same level (at-grade) and all vehicles can seamlessly move from their origin to their destination without stopping. A grid-shaped network is proposed in [34], where conventional four-leg lane intersections are replaced by a combination of suitable intersections with restrictions on the permitted lane exchanges, giving rise to paths where vehicles can safely move without stops between any two points of the grid, at the cost of an additional detour with respect to the Manhattan path, which may represent a good trade-off, especially for automated routing networks [35]. An alternative design is obtained in [36], by tiling together hexagon blocks with one-way or bidirectional links, able to avoid intersection conflicts between any nonstop paths.

In this context, a conflict-free routing system based on grid networks with alternating one-way lanes and no detour from nominal shortest paths is presented in [37]: platoons, each representing a virtual sequence of non-conflicting vehicles running on the same lane, are scheduled on a regular basis (rhythm), in such a way that, in each moment in time, just one virtual platoon crosses an intersection; each (real) vehicle is scheduled to join a synchronized nonstop sequence of virtual platoons to cover a Manhattan path from its origin to its destination. Notice that, due to the limited length of virtual platoons, a vehicle may need to wait at the border of the grid before joining the first platoon and proceeding to its destination, so that the problem is to optimize the vehicle entry times, which is modelled and solved in [37] with an integer linear programming formulation.

Even if the literature presented above shares common features with FQRP-G, it presents significant limitations in the scope of our research. In fact, as already observed, the reviewed routing algorithms may entail delays during the execution of the routes, as well as deviations from Manhattan paths. Even conflict-free routing systems involve either detours from optimal paths (like, e.g., [34]) or delays at the beginning of the schedules (like, e.g., [37]). As a consequence, the routes provided by previous methods are, in general, worse than the ones expected from the solution of FQRP-G, since we are looking for algorithms able to avoid collisions and deadlocks while preserving shortest paths with no initial nor intermediate delays. Moreover, under the hypothesis on the distribution of vehicles origins and destinations given in Section 1, the goal of FQRP-G is to minimize the grid size, which, instead, is given and fixed in previous literature, where different metrics are optimized.

Concerning works involving grid networks and nonstop collision-free routing on Manhattan paths and, hence, more strictly related to FQRP-G, a first heuristic approach is proposed in [38], where collision avoidance is guaranteed by one-way horizontal lanes and by prioritization of horizontal moves, based on the distance of a vehicle from its destination: the number of required horizontal lanes is equal to the number of vehicles, in the worst case, and smaller on average. Improved upper bounds and heuristic algorithms for FQRP-G are discussed in [7,39,40], based on the analysis of the potential conflicts arising between vehicles, and the related properties. In particular, thanks to theoretical results derived under the one-way lanes hypothesis and, as far as [7] is concerned, by restricting Manhattan paths to those containing only one horizontal leg, the number of required horizontal lanes is limited by roughly the number of vehicles divided by four, using the heuristic proposed in [7], whereas the bound claimed by the authors of [40] has to be amended, as observed in [39]. The results presented in [7,38] that are relevant for the analysis proposed in our work, will be reviewed in Section 3.

## 3. Methodology: A Mathematical Formulation

In this section, we describe a mathematical model for FQRP-G, based on a graph representation. It will be used to introduce notation and to review some relevant properties presented in the literature. By exploiting such properties, we then propose a mathematical programming formulation of FQRP-G, which will be the base for the exact approach proposed in this work.

### 3.1. Graph Model and Notation

**Figure 1:**
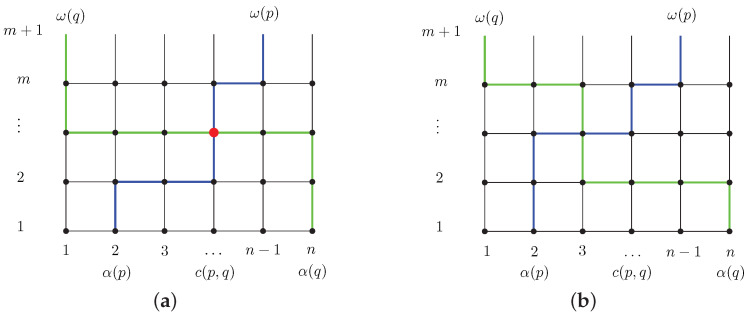
A sample grid graph with two conflicting vehicles routed on: (**a**) paths colliding in the red node; (**b**) collision-free paths.

The analysis and the development of solution methods for FQRP-G starts from modelling the grid network as an undirected grid graph G=(N,E): the set *N* contains the vertices, each corresponding to the intersection of one horizontal and one vertical lane, and the set *E* contains the lane segments, each connecting two consecutive nodes on the same horizontal or vertical lane (see Figure 1a).

We denote with *n* the number of vertical lanes (or *columns*) and with *m* the number of horizontal lanes (or *rows* or *levels*) in the grid. Columns are numbered from left to right from 1 to *n*, rows from bottom to top from 1 to *m*, so that each node representing the intersection between column *i* and row *j* is identified by the pair (i,j). Without loss of generality, we consider vehicle origins located at the bottom of the grid, in row 1, and, thus, vehicle destinations at the top of it, in row *m*. Moreover, the dummy level m+1 in Figure 1 at the top of the grid, simply represents the vehicle exit points from the grid. Let α(k) denote the starting column of vehicle *k* and ω(k) its destination column.

All vehicles start at the same time and, according to the FQRP-G definition, they never stop until they reach their destination. We recall that the time to cross an edge is constant and the same, for every edge and vehicle, and we can take it as the unit time. We can thus assume that the time is discrete and that, at each moment in time, each vehicle has to make a move, either vertically or horizontally.

We recall that, for the sake of efficiency, each vehicle has to reach its final destination using a *Manhattan path* on the grid, that is a (shortest) path that does not contain moves in opposite directions. Moreover, in order to avoid collisions, two different vehicles cannot use the same edge, or be in the same node, at the same time. In particular, no two vehicles can start from the same position, nor can share the same final position.

We also say that two vehicles *i* and *j* are in (or have) an *edge* (resp. a *node*) *conflict* between each other, if there exists a Manhattan path $\pi_{i}$ of *i* and a Manhattan path $\pi_{j}$ of *j* that use one same edge (resp. node) at the same time. If $\pi_{i}$ and $\pi_{j}$ are chosen, then a *collision* between *i* and *j* occurs. For example, vehicles *p* and *q* in Figure 1 have a node conflict, as shown, e.g., by the two Manhattan paths depicted in Figure 1a, that use the same node at time 4. Clearly, since we search for a set of pairwise collision-free paths, a solution to FQRP-G is feasible if and only if it does not contain any such a pair of paths. With reference to the example of Figure 1, notice that the node conflict between *p* and *q* can be avoided by, e.g., choosing the two Manhattan paths of Figure 1b.

We can divide the vehicles into three sets, *S*, *R* and *L*, in the following way:

S={k:ω(k)=α(k)},

R={k:ω(k)>α(k)},

L={k:ω(k)<α(k)}.

Vehicles belonging to *S* have to proceed *straight* to their final destination, and they have no conflict with other vehicles.

Vehicle $k_{1}$ belonging to *R* will have to make $\omega \left(k_{1}\right)-\alpha \left(k_{1}\right)$ horizontal moves to the *right* and may have conflicts with vehicles belonging to *L*.

Vehicle $k_{2}$ belonging to *L* will have to make $\alpha \left(k_{2}\right)-\omega \left(k_{2}\right)$ horizontal moves to the *left* and may have conflicts with vehicles belonging to *R*.

Since, for each vehicle belonging to *S*, there is only one Manhattan path, we have to choose a path only for vehicles in *R* or *L*.

In the proposed modelling framework, the FQRP-G objective can be stated as follows: we want to find the minimum number of levels necessary for all the vehicles to complete all horizontal moves before reaching their final destination column without collisions. A conflict between two vehicles can exist only if one of them belongs to *R* and the other to *L*. Moreover, since we consider nonstop Manhattan paths, further necessary conditions can be established. To this end, given vehicles $k_{1}$ and $k_{2}$, let $c\left(k_{1},k_{2}\right)=\left\lfloor \alpha \left(k_{1}\right)+\alpha \left(k_{2}\right)\right\rfloor /2$.

An edge conflict between vehicles $k_{1}$ and $k_{2}$ exists if and only if the pair $\left(k_{1},k_{2}\right)$ belongs to the set

(1) $$\begin{array}{c} C_{odd}=\{\left(k_{1},k_{2}\right)\in R\times L:\;\alpha \left(k_{1}\right)<\alpha \left(k_{2}\right),\;\alpha \left(k_{1}\right)+\alpha \left(k_{2}\right)\;\mathrm{is}\;\mathrm{odd},\\ \;\omega \left(k_{1}\right)\geq c\left(k_{1},k_{2}\right)+1,\;\omega \left(k_{2}\right)\leq c\left(k_{1},k_{2}\right)\}. \end{array}$$

The conflict only occurs on a horizontal edge joining a node of column $c(k_{1},k_{2})$ with a node of column $c(k_{1},k_{2})+1$. To avoid collisions related to edge conflicts between vehicles $k_{1}$ and $k_{2}$ with $\left(k_{1},k_{2}\right)\in C_{odd}$, vehicles $k_{1}$ and $k_{2}$ have to cross the space between columns $c(k_{1},k_{2})$ and $c(k_{1},k_{2})+1$ at different levels.

A node conflict between vehicles $k_{1}$ and $k_{2}$ exists if and only if the pair $\left(k_{1},k_{2}\right)$ belongs to the set

(2) $$\begin{array}{c} C_{even}=\{\left(k_{1},k_{2}\right)\in R\times L:\;\alpha \left(k_{1}\right)<\alpha \left(k_{2}\right),\;\alpha \left(k_{1}\right)+\alpha \left(k_{2}\right)\;\mathrm{is}\;\mathrm{even},\\ \omega \left(k_{1}\right)\geq c\left(k_{1},k_{2}\right),\;\omega \left(k_{2}\right)\leq c\left(k_{1},k_{2}\right)\}. \end{array}$$

The conflict only occurs on a node of column $c(k_{1},k_{2})$.

Node conflicts can be further classified as (see [7]):

- *B-conflict*, if $\omega \left(k_{1}\right)>c\left(k_{1},k_{2}\right)$ and $\omega \left(k_{2}\right)<c\left(k_{1},k_{2}\right)$,
- *C-conflict*, if either $\omega \left(k_{1}\right)=c\left(k_{1},k_{2}\right)$ or $\omega \left(k_{2}\right)=c\left(k_{1},k_{2}\right)$.

To avoid collisions related to B-conflicts between vehicles $k_{1}$ and $k_{2}$ such that $\left(k_{1},k_{2}\right)\in C_{even}$, the set of nodes in column $c(k_{1},k_{2})$ visited by vehicle $k_{1}$ has to be disjoint from the set of nodes in the same column visited by vehicle $k_{2}$.

Consider now vehicles $k_{1}$ and $k_{2}$ such that $\left(k_{1},k_{2}\right)\in C_{even}$, subject to a C-conflict, and assume that $\omega \left(k_{2}\right)=c\left(k_{1},k_{2}\right)$. In this case, any Manhattan path of vehicle $k_{2}$ reaches column $c(k_{1},k_{2})$ and then proceeds with vertical steps only, remaining in such column. Therefore, vehicle $k_{1}$ needs to visit and leave column $c(k_{1},k_{2})$ before $k_{2}$ reaches this column. Hence, as observed by the authors of [7], to avoid collisions related to such C-conflict, it is necessary and sufficient that vehicle $k_{1}$ leaves column $c(k_{1},k_{2})$ on a lower level than that on which vehicle $k_{2}$ reaches it.

Given two vehicles $k_{1}$ and $k_{2}$ subject to a C-conflict, we say that *$k_{2}$ has a C-conflict with $k_{1}$* if $\omega \left(k_{2}\right)=c\left(k_{1},k_{2}\right)$ and, vice versa, *$k_{1}$ has a C-conflict with $k_{2}$* if $\omega \left(k_{1}\right)=c\left(k_{1},k_{2}\right)$. Such relation is not symmetric: if $k_{2}$ has a C-conflict with $k_{1}$, then $k_{1}$ does not have a C-conflict with $k_{2}$. Furthermore, if $k_{2}$ has a C-conflict with $k_{1}$, then it cannot have any other C-conflict with any vehicle distinct from $k_{1}$.

### 3.2. Review of Relevant Previous Results

Andreatta et al. in [38] consider FQRP-G and propose a heuristic dispatching algorithm (DA) to solve it. The algorithm incrementally builds vehicle routes and its underlying idea is to give priority to the horizontal movement of the vehicles with higher numbers of remaining horizontal steps. DA provides collision-free Manhattan paths and its computational complexity is $O(n^{2})$. As observed in [38], the route generated by DA for any vehicle is, by construction, a *simple* Manhattan path, i.e., a Manhattan path such that all its horizontal moves are performed on one level only. Moreover, no level contains horizontal moves in opposite directions, that is, grid rows corresponds to one-way horizontal lanes. Concerning the objective function value, the number of necessary levels, i.e., the number of levels at which at least one vehicle moves horizontally, is bounded by the number of vehicles, hence by *n*, in the worst case, even if it can be significantly smaller for specific FQRP-G instances.

The minimum number of levels that ensures the existence of collision-free routes in any instance of FQRP-G for a given *n*, has been deeply investigated by Cenci et al. in [7]. They tackle FQRP-G defining *C-conflict paths*, i.e., sequences of vehicles such that each vehicle in the sequence has a C-conflict with the following one (we recall that the definition of C-conflict is not symmetric). They prove that the length of the longest C-conflict path that can be observed in any instance of FQRP-G on a grid with n≥3 columns is equal to

(3) $$1+\left\lfloor \frac{n-1}{4}\right\rfloor .$$

Then they assume that only simple Manhattan paths are feasible and that each grid level allows movements in one direction only (one-way horizontal lanes). These conditions exclude collisions related to edge and B-conflicts and restrict the attention to C-conflicts. Under such hypotheses, Ref. [7] proves that, for n≥3,

(4) $$m^{*}=3+\left\lfloor \frac{n-1}{4}\right\rfloor$$

is the number of levels of the grid that guarantees the existence of a feasible solution to every instance of FQRP-G. As a minor result, they provide an algorithm (called *CaR*) to solve any instance of FQRP-G on a grid graph $n\times m^{*}$ with time complexity $O(n^{3})$, thus showing that $m^{*}$ horizontal lanes are also sufficient.

An important byproduct of the research in [7], which will be relevant for the analysis proposed in this paper, is the definition of the *C-conflict directed graph* F=(V,A), where *V* is the set of vehicles and *A* is the set of arcs, defined as follows: given two vehicles $k_{1}$ and $k_{2}$, $(k_{1},k_{2})\in A$ if and only if $k_{2}$ has a C-conflict with $k_{1}$. In other words, arcs are associated with C-conflicts: arc $\left(k_{1},k_{2}\right)$ means that the route of $k_{1}$ must be strictly below the route of $k_{2}$ in column $\omega (k_{2})$ of graph *G*. Notice that the definition of C-conflict directed graph given above slightly differs from the one in [7], as the arc orientation is opposite. This allows us to restate one of the results in [7] as follows, and to provide a formal proof (recall that an arborescence is a directed rooted tree such that the path from the root to any other node is unique).

**Proposition** **1** ([7])**.** *The C-conflict directed graph F is a forest of arborescences.*

**Proof.**  Suppose that the directed graph *F* contains a cycle $k_{1},\;k_{2},\;\ldots ,\;k_{c},\;k_{c+1}=k_{1}$. For any pair of consecutive vehicles in the cycle, $k_{i}$ and $k_{i+1}$, vehicle $k_{i+1}$ has a C-conflict with vehicle $k_{i}$ by the definition of arc in *F*. It follows that the number of horizontal steps in the route of $k_{i}$ (equal to $|\omega \left(k_{i}\right)-\alpha \left(k_{i}\right)|$) is strictly greater than the number of horizontal steps in the route of $k_{i+1}$, for any i=1,…,c. However, this contradicts the fact that $k_{1}=k_{c+1}$. Therefore, the directed graph *F* does not contain cycles. Furthermore, as each vehicle can have a C-conflict with at most one other vehicle, each node of *F* has at most one entering arc, and thus the path from the root to any node is unique. It follows that *F* is a forest of arborescences. □

### 3.3. A Mathematical Programming Formulation

In this section, we propose a mathematical programming formulation of FQRP-G. Mathematical programming is a well-known operations research tool to model and solve optimization problems. A mathematical programming model defines numerical decision variables and, based on these variables, an objective function, and a system of equations and inequalities (constraints): the objective function is the quantity to be maximized or minimized, whereas the constraints define the set of feasible solutions. Solving a mathematical programming model means finding a solution that satisfies all the constraints and optimizes the value of the objective function. Integer linear programming formulations are mathematical programming models where the objective, as well as the constraints, are linear functions of the decision variables, and (some of) the variables are restricted to assume integer values only. There is no known polynomial-time algorithm to solve general integer linear programming models (indeed, this is an NP-hard problem [41]), but standard techniques are available, like, e.g., branch and bound or cutting planes algorithms and further improvements (see, e.g., [42,43]), whose running time is expected to grow exponentially with the size (number of variables and constraints) of the formulation. However, these techniques are implemented by state-of-the-art solvers, which provide effective off-the-shelf tools to solve optimization problems formulated as integer linear programming models in a wide range of applications, including collision-free network design and routing (e.g., [25,37]), at least for moderate-size instances.

The integer linear programming formulation we propose for FQRP-G is based on network flow models (see, e.g., [26]). For each vehicle k∈R, let us introduce the following binary variables $x_{ijk}^{v}$ and $x_{ijk}^{h}$, representing decisions about vertical and, respectively, horizontal moves:

- variable $x_{ijk}^{v}$ is equal to 1 if the edge joining nodes (i,j) and (i,j+1) belongs to the chosen shortest path of vehicle *k* (and 0 otherwise); these variables are defined for every triplet i,j,k such that α(k)≤i≤ω(k) and 1≤j≤m;
- variable $x_{ijk}^{h}$ is equal to 1 if the edge joining nodes (i,j) and (i+1,j) belongs to the chosen shortest path of vehicle *k* (and 0 otherwise); these variables are defined for every triplet i,j,k such that α(k)≤i≤ω(k)−1 and 1≤j≤m.

For each vehicle k∈L, let us introduce the following binary variables $y_{ijk}^{v}$ and $y_{ijk}^{h}$, which are the homologous of *x* variables above:

- variable $y_{ijk}^{v}$ is equal to 1 if the edge joining nodes (i,j) and (i,j+1) belongs to the chosen shortest path of vehicle *k* (and 0 otherwise); these variables are defined for every triplet i,j,k such that ω(k)≤i≤α(k) and 1≤j≤m;
- variable $y_{ijk}^{h}$ is equal to 1 if the edge joining nodes (i,j) and (i−1,j) belongs to the chosen shortest path of vehicle *k* (and 0 otherwise); these variables are defined for every triplet i,j,k such that ω(k)+1≤i≤α(k) and 1≤j≤m.

We remind that the dummy level m+1 represents the vehicle exit points from the grid. Therefore, variables $x_{imk}^{v}$ or $y_{imk}^{v}$, just above defined, are equal to 1 if vehicle k from the top of column *i* moves out of the grid.

Finally, let us introduce a variable *z*, whose meaning is the highest level where a horizontal move takes place.

**Figure 2:**
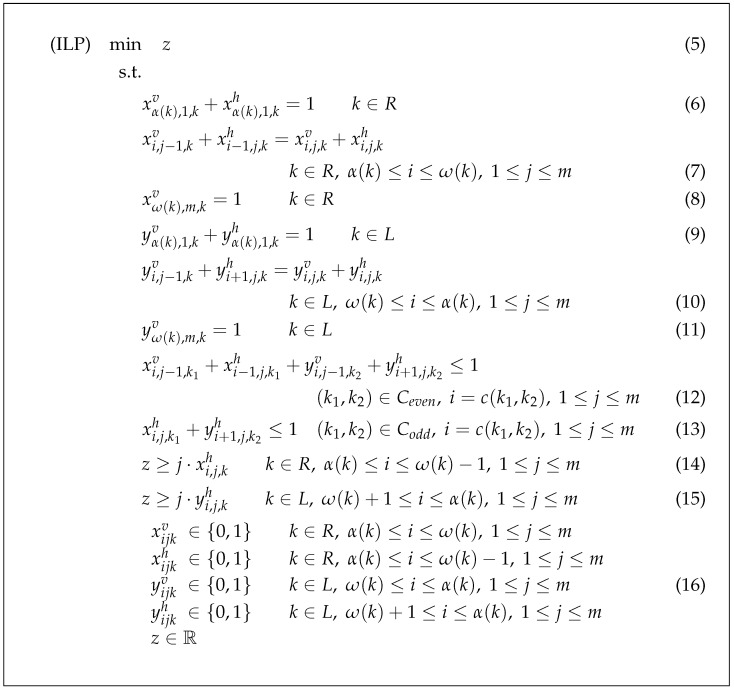
The integer linear programming formulation of FQRP-G (ILP).

The proposed integer linear programming formulation of FQRP-G (ILP) is reported in Figure 2. As from the objective function (5), we are interested in minimizing *z*, i.e., we want to find the minimum number of levels necessary for all the vehicles to complete all horizontal moves before reaching their final destinations.

Overall, constraints (6)–(11) guarantee that, for each vehicle, the edges associated with variables that take value 1 provide a (shortest) Manhattan path: constraints (6)–(8) are devoted to vehicles in *R* whereas constraints (9)–(11) to vehicles in *L*. Constraints (6) and (9) require that the route of vehicle *k* starts at position (α(k),1) with either a horizontal step or a vertical one. Then, equalities (7) and (10) state flow conservation, that is: if vehicle *k* reaches node (i,j) (either with a vertical or a horizontal move, see the left-hand side), then *k* must perform either a vertical or a horizontal move starting from the same node (see the right-hand side). For the sake of clarity, notice that constraints are stated regardless of the fact that, for some boundary values of indexes *i* and *j*, some of the variables involved in (7) and (10) are not defined and must be replaced by 0. In the definition of constraint (7) for k∈R, this happens for the following variables: (i) $x_{i-1,j,k}^{h}$, if i=α(k); (ii) $x_{i,j,k}^{h}$, if i=ω(k) and (iii) $x_{i,j-1,k}^{v}$, if j=1. With similar arguments, in the definition of constraint (10) for k∈L, the following variables must be replaced by 0: (i) $y_{i+1,j,k}^{h}$, if i=α(k); (ii) $y_{i,j,k}^{h}$, if i=ω(k) and (iii) $y_{i,j-1,k}^{v}$, if j=1. Equalities (8) and (11) require that the route of vehicle *k* reaches the top of the destination column with a vertical step.

After the observation that defines sufficient conditions to avoid collisions related to node conflicts (see Section 3.1), such collisions are avoided by constraints (12): they state that at most one of the two vehicles involved in a given conflict can reach, with either a vertical or a horizontal move, the potential collision position, i.e., the same row in the conflict column. Even for these constraints, boundary index values are solved by replacing $x_{i,j-1,k_{1}}^{v}=y_{i,j-1,k_{2}}^{v}=0$ in case j=1. Even according to the sufficient conditions stated in Section 3.1, constraints (13) prevent collisions related to edge conflicts, since they exclude routes where two vehicles in such a conflict move between the interested columns at the same level.

Variable *z* is linked to variables *x* and *y* through (14) and (15), stating that at least *j* levels are required if at least one horizontal move takes place at row *j*.

The objective is to find the minimum of *z*. Finally, constraints (16) set variables *x* and *y* as binary and variable *z* real. Notice that *z* integrality follows, by (14), (15) and the objective function (5), from integrality of $x^{h}$ and $y^{h}$. Moreover, even $x^{v}$ and $y^{v}$ could be defined as continuous, since their integrality follows from the one of $x^{h}$ and $y^{h}$ by (6), (7), (9) and (10).

We remark that the proposed ILP model describes, for each vehicle, a static flow on the grid network, since no time component is required to define both the decision variables and the constraints, in view of the conditions devised in Section 3.1 to bound the set of possible collision points. This is different from the mathematical programming formulations presented in, e.g., [25], where flows are defined on a time-expanded network, or [37], where the impact of the flow on different rhythmic routing intervals has to be considered. As a consequence, the size of ILP, in terms of the number of both variables and constraints, is considerably smaller than the corresponding formulations presented in previous literature, with benefits for the required solution time.

## 4. Heuristics

The mathematical model presented in Section 3 can be solved through off-the-shelf solvers for mixed-integer linear programming to obtain an optimal solution for a given FQRP-G instance, i.e., the minimum number of rows that allows collision-free nonstop Manhattan paths to route vehicles on, together with the paths themselves. However, due to the computational complexity of integer linear programming, we expect that the efficiency of the model, in terms of time to obtain the optimal solution, degrades with the size of the instance to handle, as in fact our computational experiments, presented in Section 5, ascertain. We thus propose two heuristics to solve FQRP-G. The first one, called Heuristic A, is a reinterpretation of the CaR algorithm given by Cenci et al. in [7], but it is much simpler and improves the computational complexity, as will be stated in Proposition 4. It uses the C-conflict directed graph to generate vehicle routes that are simple Manhattan paths and it is able to always provide a feasible solution to FQRP-G. The second one, called Heuristic B, is more flexible in choosing Manhattan paths and attempts to determine the vehicle routes by giving priority to the horizontal moves of vehicles ranked on the basis of measures obtained from the C-conflict directed graph.

### 4.1. Heuristic A

Heuristic A is based on the C-conflict directed graph *F*. As from Proposition 1, each connected component of *F* is an oriented arborescence. Any such arborescence has a root, and its nodes can be partitioned according to their depth. The root has zero depth. The depth of any node is equal to the length of the unique path in *F* from the root to that node. For each node, we also define its height as the length of the longest path in *F* from that node to any of the leaves. The height of a connected component is equal to the length of the longest path from its root, i.e., the height of the root itself. Vehicles in *S*, as well as any vehicle that is not involved in C-conflicts, are isolated nodes in *F*, and have both height and depth equal to 0. The root of any non-trivial arborescence is either in *L* or in *R*.

We now state Heuristic A and, then, we discuss its correctness and properties. Given an instance of FQRP-G, in terms of the number of columns *n*, set of vehicles and related origins α and destinations ω, Heuristic A runs through the following steps:

1. Partition the set of vehicles into *S*, *R* and *L* and build the C-conflict directed graph *F*. Assume, without loss of generality, that a connected component with maximal height has the root in *R* (the case in *L* is similar).
2. Let *p* be any vehicle in a connected component rooted in *R*, and let $l_{p}$ be its depth in *F*. The route of vehicle *p* is as follows: move vehicle *p* vertically on column α(p) to reach level $l_{p}+1$, and then horizontally on level $l_{p}+1$ until column ω(p); then move it vertically to its final destination.
3. Let *q* be any vehicle in a connected component rooted in *L*, and let $l_{q}$ be its depth in *F*. The route of vehicle *q* is as follows: move vehicle *q* vertically on column α(q) to reach level $l_{q}+2$, and then horizontally on level $l_{q}+2$ until column ω(q); then move it vertically to its final destination.
4. The route of vehicles in *S* contains vertical steps only.

The following proposition shows that Heuristic A always provides a feasible solution.

**Proposition** **2.** *The vehicle routes given by Heuristic A are nonstop collision-free simple Manhattan paths.*

**Proof.**  In the output of Heuristic A, all the horizontal moves performed at any level have the same direction. Indeed, each path in *F* is a C-conflict path and, hence, it alternates vehicles in *R* and in *L*. If follows that, under the assumption that the maximum height is related to an arborescence rooted in *R* (the case in *L* is similar) all vehicles in *R* move horizontally on an odd row, and all vehicles in *L* on an even row. This corresponds to having one-way horizontal lanes, which prevents collisions related to edge conflicts from occurring. We observe that, trivially, Heuristic A outputs simple Manhattan paths, as each vehicle performs consecutively all its horizontal moves on the same level. This, together with one-way lanes, avoids collisions related to B-conflicts.

For each pair of vehicles *p* and *q* such that *q* has a C-conflict with *p*, the directed graph *F* contains the arc (p,q), and $l_{q}=l_{p}+1$ holds. Therefore, vehicle *p* performs its horizontal moves on a lower level than *q* does, and the route of *p* is below the one of *q* in column ω(q), as required to avoid collisions related to C-conflicts. □

Notice that, if Heuristic A is applied to a single connected component of *F*, then the number of grid levels used by the output solution is equal to one plus the height of that component. Therefore, given any instance, the number of grid levels needed by Heuristic A is equal the height of the highest connected component of *F*, added by 2. The term “+2” comes from the case in which the forest *F* contains two (or more) highest components, of which, one rooted in *R* and another in *L*. This proves the following

**Proposition** **3.** *Given an instance of FQRP-G and its C-conflict direct graph, let ${\bar{m}}_{R}$ and ${\bar{m}}_{L}$ be the maximum height of an arborescence rooted in R and, respectively L. The number of levels required by Heuristic A is $\max\{{\bar{m}}_{R},{\bar{m}}_{L}\}+a$, where a=1 if ${\bar{m}}_{R}\neq {\bar{m}}_{L}$, a=2 otherwise.*

The number of required levels is equal to the one stated for algorithm CaR proposed by Cenci et al. in [7]: as shown in [7], CaR optimally solves FQRP-G if the set of vehicle routes is restricted to simple Manhattan paths and under the hypothesis of one-way horizontal lanes. We thus have the following

**Corollary** **1.** *Heuristic A finds the optimal solution of FQRP-G restricted to simple Manhattan paths and one-way horizontal lanes.*

In fact, as already stated above, Heuristic A is a reinterpretation of the CaR algorithm that improves its computational complexity (we recall that CaR runs in $O{\left(n\right)}^{3}$).

**Proposition** **4.** *Given an instance of FQRP-G on a grid network with n columns, the computational complexity of Heuristic A is O(n).*

**Proof.**  In order to detect all C-conflicts, O(n) calculations are sufficient. Indeed, for any vehicle k∈R (resp. in *L*), we only have to check if there is another vehicle moving from position (2ω(k)−α(k),1) and having its destination on the left (resp. on the right) of column ω(k); in such case, vehicle *k* has a C-conflict with the other vehicle. All the arcs of *F* can be thus detected in at most *n* (a bound on the number of vehicles) operations, and *F* built in O(n). All the data required by Heuristic A can be collected during a depth-first visit of *F*, which allows computing the depth and the height of any node in O(n). This shows that Step 1 takes O(n) operations. Concerning Steps 2 to 4, they simply assign the horizontal level to each vehicle, which can still be done in O(n). □

### 4.2. Heuristic B

Heuristic B aims at calling non-simple Manhattan paths conveniently into play. The underlying idea is to find an appropriate order of the vehicles and, then, to sequentially route each vehicle on the “lowest” Manhattan path possible, i.e., a Manhattan path obtained by choosing a horizontal step whenever this is compatible with previously assigned paths.

Vehicles are sorted according to a measure of how critical it is to route them. For example, an order of the vehicles could provide a feasible set of routes only if, for any pair of vehicles $k_{1},k_{2}$ such that $k_{2}$ has a C-conflict with $k_{1}$ on column $\omega (k_{2})$, vehicle $k_{1}$ precedes $k_{2}$ in the order, since otherwise $k_{2}$ would have precedence in the horizontal move to reach the conflict column and stay below $k_{1}$ on it, which means that the C-conflict cannot be resolved (see Section 3.1). It follows that vehicles belonging to a C-conflict path should be sorted in the increasing order of their depth in the C-conflict directed graph *F*, which again plays an important role in prioritizing vehicles. We also observe that, in general, the assigned Manhattan paths are not simple and each level can be run in opposite directions; therefore, both edge conflicts and B-conflicts may actually generate collisions and have to be taken into account.

For each vehicle *k*, the following measures are considered:

- $l_{k}$: the depth of *k* in *F*;
- $\gamma_{k}$: length of the longest C-conflict path *k* belongs to. Notice that, if $l_{k}$ and $h_{k}$ are, respectively, the depth and the height of *k* in *F*, $\gamma_{k}=l_{k}+h_{k}$, and, in particular, $\gamma_{k}=0$ if *k* is not involved in any C-conflict;
- $\delta_{k}$: overall number of conflicts *k* is involved in;
- $\rho_{k}$: number of edge conflicts *k* is involved in.

Given an instance of FQRP-G, in terms of number of columns *n*, set of vehicles and related origins α and destinations ω, Heuristic B runs through the following steps:

1. Compute an upper bound $\bar{m}$ on the number of required levels (it can be simply equal to the number of vehicles, or it can be obtained by running Heuristic A).
2. Build the C-conflict directed graph *F* and, for each vehicle *k*, compute $\gamma_{k}$, $l_{k}$, $\delta_{k}$ and $\rho_{k}$;
3. Sort vehicles according to any order such that they appear by non-decreasing $l_{k}$;
4. For each vehicle *k* in the determined order, assign *k* to the “lowest” available Manhattan path, as recursively defined by the following rule (given for the case k∈R, the case k∈L∪S is similar):
  (a) let (i,j) be the actual position of vehicle *k* in the grid (initially set to (α(k),1));
  (b) if i=ω(k) and $j=\bar{m}$, then output “feasible path for *k* found” and consider the next vehicle;
  (c) if i=ω(k) and all vehicles up to *k* in a C-conflict path are not involved in further conflicts, then *k* performs a vertical move;
  (d) otherwise, if i≠ω(k), then check if the horizontal move to node (i+1,j) involves any collision with previously assigned paths (this could be related to a node-conflict if, after a unit of time, another vehicle will be in node (i+1,j), or an edge conflict if another vehicle is performing the opposite move from (i+1,j) to (i,j) at the same time); if the answer is “no conflict”, then *k* performs the horizontal move to node (i+1,j);
  (e) otherwise, check if the vertical move to (i,j+1) involves any conflict with previously assigned paths (this could be a node-conflict if, after a unit of time, another vehicle will be in node (i,j+1)); if the answer is “no conflict”, then *k* vertically moves to node (i,j+1);
  (f) otherwise, output “no feasible path for *k* found” and stop.

With reference to Step 4c, we remark that, since vehicles are sorted by non-decreasing $l_{k}$, collisions related to C-conflicts are avoided, as for any arc $\left(k_{1},k_{2}\right)$ of *F*, the path of vehicle $k_{1}$ is set before the path of $k_{2}$. These are the only collisions associated with vertical moves on the destination columns, so that, in the case specified by Step 4c, checking their occurrence is redundant.

While, in the above case, C-conflicts are solved by appropriately ordering the vehicles in the first phase of the algorithm, remaining node-conflicts and edge conflicts are tentatively solved during Steps 4d–4e. However, we have no guarantee to avoid related collisions and, indeed, Heuristic B may get stuck if both horizontal and vertical moves of a vehicle at a given node are forbidden. Nevertheless, if Heuristic B is successful, the required number of levels is not bounded from below by the length of the longest C-conflict path, as it is the case for Heuristic A: we thus aim to empirically evaluate the probability of getting stuck and, if this is not the case, the ability of Heuristic B to provide better results than Heuristic A.

The actual performance of Heuristic B depends on the specific sorting adopted by Step 3. We propose two alternatives giving rise to:

- Heuristic B1: vehicles are sorted in lexicographic order by decreasing $\gamma_{k}$, increasing $l_{k}$, decreasing $\delta_{k}$ and decreasing $\rho_{k}$;
- Heuristic B2: vehicles are sorted in lexicographic order by increasing $l_{k}$, decreasing $\gamma_{k}$, decreasing $\delta_{k}$ and decreasing $\rho_{k}$.

We now discuss the computational complexity of Heuristic B.

**Proposition** **5.** *Given an instance of FQRP-G on a grid graph with n columns, the computational complexity of Heuristic B is $O(n^{2}\;\bar{m})$.*

**Proof.**  Step 1 to determine $\bar{m}$ can be done in O(n). The measures required by the sorting step can be computed by building and depth-first visiting the C-conflict directed graph *F*, which can be done in O(n) (as discussed in proof of Proposition 4). The sorting Step 3 takes O(nlogn). Since the number of moves in a Manhattan path is bounded by $n+\bar{m}$, and the number of vehicles by *n*, the complexity of Step 4, and of overall Heuristic B, is $O(n^{2}\;\bar{m})$. □

## 5. Results

In the previous sections, we propose the integer linear programming (ILP) formulation and three heuristics (A, B1, and B2) to solve FQRP-G. Computational experiments have been conducted with the following purposes:

- determine to what extent, in terms of instance size and required running time, ILP is able to solve FQRP-G;
- assess the quality of the solutions output by Heuristic A (which, we recall, is optimal under one-way horizontal lanes and simple Manhattan paths hypothesis) in terms of additional required levels with respect to the (unrestricted) optimal solution provided by ILP;
- estimate the success rate of Heuristics B1 and B2 and their ability to find better solutions than Heuristic A.

We recall that, as discussed in Section 2, previous literature approaches to collision-free routing problems present limitations in their application to FQRP-G, since they do not consider grid-size minimization and, moreover, they allow for space-time deviations from nonstop Manhattan paths. The heuristic algorithm DA presented in [38] is able to solve FQRP-G, however it is dominated by Heuristic A for both efficiency since DA is $O(n^{2})$ whereas Heuristic A is O(n), and effectiveness. Indeed, as observed in [38], DA returns routing schedules made of simple Manhattan paths on one-way horizontal lanes and, hence, compliant with the hypothesis of Corollary 1: as a consequence, DA cannot provide better solutions than Heuristic A, which is optimal under such restrictions.

In our experiments, we consider two benchmarks. The first one is made of random instances with 10 up to 300 columns and vehicles: in particular, 20 instances are generated for each n∈{10,25,50,75,100,150,200,300} by randomly choosing the origin and the destination columns of each vehicle. The second benchmark includes 11 ad hoc instances with 105 up to 233 columns and vehicles, created on purpose as to contain long C-conflict paths, and more than one arborescences in the related C-conflict directed graph. ILP has also been run on a third benchmark of large random instances with n∈{350,400,500}, to determine the larger size instances ILP can solve in practice.

All the tests were run on a workstation equipped with an Intel Xeon E-2176G processor with 6 cores at 3.7 GHz, and 16 GB RAM.

ILP has been solved using the Cplex 12.9.0 engine [44] with a time limit of 30 min. In order to take the number of variables and constraints of ILP, hence running times, as small as possible, we run Heuristic A (whose running time, as we will see, is negligible) and set the parameter *m* in the ILP model equal to the number of levels output by Heuristic A.

Table 1 reports the computational results given by ILP and Heuristic A on the first random benchmark. Statistics involving ILP refer to tests on 10 out of 20 instances available per size. The first column specifies the instance size. The average, minimum and maximum number of levels used by ILP are reported in Columns 2 and 3. ILP running times, whose average (in seconds) appears in Column 4, are below the time limit in every instance and, therefore, data in Columns 2 and 3 refer to proven optimal values. Columns 5–8 are related to Heuristic A and give respectively: the average number of levels used by its solutions, the relative percentage error with respect to the optimal ILP value, the minimum and the maximum number of levels required by all the obtained solutions, and the maximum absolute gap between the number of levels used by the solutions of Heuristic A and the corresponding optimal values. Running times of Heuristic A are not specified as they are negligible (always fairly less than 1 ms).

The computational results given by Heuristics B1 and B2 on the first random benchmark appear in Table 2. The percentage of instances where Heuristic B1 has been able to find a feasible solution (success rate) is reported in Column 2. Columns 3 to 6 refer to these successful instances and report: the average, minimum and maximum number of levels required by B1 (Columns 3 and 5 respectively); the average per cent error in the number of levels used by B1 with respect to the optimal value output by ILP (Column 4); the maximum absolute gap between the number of levels used by B1 and the optimal values (Column 6). Always referring to successful instances for B1, Column 7 compares the performances of Heuristic B1 versus Heuristic A, reporting the percentages of successful instances in which B1 uses less (win) or more (lose) levels than A. Columns 8 to 13 report the same information for Heuristic B2. Again, statistics involving ILP refer to tests on 10 out of 20 instances available per size.

ILP was able to find the optimal solution of all the instances within the time limit, and running times are consistently less than a few seconds up to 100 vehicles. For larger sizes, running time grows almost exponentially, as expected. Indeed, we performed a further test of ILP on the third benchmark, observing that only four out of ten cases with n=350 (and no other larger instances) are solved to optimality. In the remaining cases with n=350, ILP always finds feasible solutions whose difference with respect to the best available lower bound (optimality absolute gap) is 2.5 levels on average (maximum 4). The success rate on 400 columns instances is 90%, i.e., ILP finds feasible (even if not provably optimal) solutions for 9 out of 10 instances, with an optimality absolute gap of 3 levels on average (maximum 4). For n=500, the success rate is 60%, with optimality absolute gap of 3.5 levels (maximum 4). We also observe that, as far as the third benchmark is concerned, the number of required horizontal lanes never exceeds 6 in the proposed feasible solutions.

Heuristic A is extremely fast, and, as from Table 1, it finds solutions that, even for larger random instances, take no more than 5 levels and at most 2 additional horizontal lanes with respect to the optimal values.

Running times of Heuristics B1 and B2 are negligible as well (always less than $10^{-2}$ s), however, their performance is poor. Both B1 and B2 get stuck in all the instances with 75 or more vehicles. The success rate is acceptable only for very small instances and just, in a few cases, B1 and B2 are able to improve over Heuristic A (with B2 showing slightly better results than B1). Summarizing the overall performance of Heuristic A on random instances is by far better than B1 and B2.

As observed above, the number of levels required by Heuristic A is very small in random instances. However, we recall that it is strictly connected to the length of the longest C-conflict path in the instance, so that, according to Equations (3) and (4), instances exist where the collision-free paths outputted by Heuristic A need more than a few horizontal lanes to be seamlessly operated. Therefore, we consider the second benchmark of ad hoc generated instances containing long C-conflict paths, more than one arborescence in the C-conflict directed graph, and further edge conflicts and B-conflicts between vehicles in the same or different arborescences. Table 3 reports the related computational results, showing a row for each instance. The number of vehicles and the length of the longest observed C-conflict path appears in Columns 1 and 2. Columns 3 and 4 give the number of levels required by the solution of Heuristic A and by the optimal solution of the ILP model, respectively. The ILP model running times, in seconds, are listed in the last column (the table does not show Heuristic A running times, since they are always less than $10^{-3}$ s). Results for Heuristics B1 and B2 are not reported, since they always fail in providing feasible routes.

ILP solves all the instances of the second benchmark to optimality, still providing routes that can be operated on a few (at most four) horizontal lanes. It is thus self-evident that Heuristic A is not appropriate to solve FQRP-G on these ad hoc instances, as it needs many more levels with respect to the optimal solution. Indeed, performing horizontal steps on more than one level is crucial, in presence of long chains of C-conflicts, to save levels. However, the ad hoc instances in the third benchmark do not appear much harder to be solved with ILP in terms of computational time.

## 6. Discussion

The methods presented in the previous sections allow us to find provably optimal or heuristic solutions to FQRP-G. The problem is relevant for the design and the operation of automated transportation systems where the routing network consists of intersecting horizontal and vertical lanes, vehicles move between opposite sides (e.g., from bottom to top) and a network of sensors supports safe and efficient operations: port container terminals, automated warehouses, train terminals, etc., are some significant examples that can be approximated by such routing networks. By solving FQRP-G, the number of horizontal lanes and a set of routes is determined that can be seamlessly operated without intermediate stops nor deviations from static shortest paths (efficient routes) and without any collision (safe routes). In real-time, the sensing network and related logic monitor the operations and manage further conflicts just in case they arise due to unpredicted events (vehicle breakdowns, network interruptions, etc.), making the overall routing system robust against disruptions, and further reducing the risk of collisions.

While the number of vertical lanes is often determined by the facility layout, the number of required horizontal lanes should be carefully dimensioned at the design phase, in order to minimize the cost of the underlying transportation and sensing infrastructure. In this work, we have devised and tested four possible approaches to solve FQRP-G and determine the minimum number of levels, given vehicles’ initial positions and final destinations: an exact method (ILP) based on solving an integer linear programming formulation of the problem by standard solvers, and three heuristics (A, B1 and B2) that prioritize vehicles based on the properties of a graph summarizing C-conflicts between vehicles.

Experiments on benchmarks of random and ad hoc instances show that, from a computational point of view, ILP is able to find the proven minimum number of horizontal lanes (with related vehicle routes) for instances of up to 300 vehicles, even if running times seem to be suitable for real-time operations of up to about 100 vehicles. For larger random instances, Heuristic A always provides, in negligible running time, feasible routes with at most two additional horizontal lanes, if compared to the optimal solutions, while heuristics B1 and B2 often fail in finding a set of non-conflicting vehicle paths.

From a network design perspective, it is interesting to notice that the optimal solution for the tested instances (up to 300 vehicles) always requires no more than 3 levels (4 in 2 out of 80 cases), thus suggesting that the size of the transportation network can be set to a relatively small number of horizontal lanes. Even more interestingly, our experimental results show that, at the cost of a few additional horizontal lanes, Heuristic A can be run to produce feasible seamless routes for the case where, due to limited computational resources, solving ILP is unpractical. Moreover, Heuristic A has the advantage of providing simple Manhattan paths that can be run on a network with one-way lanes and leads to a simpler network to design, monitor and maintain, as well as to smoother, safer and simpler routes to operate. The drawback is that the number of levels required by Heuristic A may be very large with respect to the optimal one, as our experiments on ad hoc instances show: however, such instances (with long chains of vehicles in C-conflict paths) seem to be extreme cases and, in fact, they never occurred in random experiments. Moreover, they get solved by ILP in less than 40 s, even for the larger 233 vehicles instance, with optimal solutions requiring, as for random instances, no more than four horizontal lanes. It follows that an automated grid transportation network can be conveniently designed with a relatively small number of horizontal lanes and operated through ILP or Heuristic A (depending on instance size and available computational resources), leaving to the sensor network and to the run-time collision detection and avoidance system (based, e.g., on more general methods for collision-free routing presented in literature) the rare cases where the proposed methods do not find feasible solutions to FQRP-G.

Our experiments with ILP show that a grid routing network with four horizontal lanes has always been able to accommodate routing paths according to the requirements of FQRP-G. In case an exact solution method (like ILP) is not conveniently available, the proposed heuristic would require at most five horizontal lanes in almost all of the FQRP-G instances. For the residual cases, a grid network with five horizontal lanes may not guarantee nonstop routing on Manhattan paths for all vehicles: in such events, the envisioned routing system can be integrated with state-of-the-art algorithms for multi-agent pathfinding, like the ones presented in the literature review, in order optimize any required space-time deviations from nominal shortest routes.

## 7. Conclusions

In this work, we addressed FQRP-G, where a set of vehicles has to be routed on a grid network according to a set of nonstop collision-free Manhattan paths that minimizes the overall number of required horizontal lanes. Such paths can be seamlessly operated with no further control logic for collision and deadlock detection and avoidance, leaving a sensor network as the only task to guarantee safe operations against an unexpected vehicle or infrastructure disruptions during the real-time execution.

We have presented an integer linear programming formulation of FQRP-G, called ILP, and three heuristics, showing that:

- a theoretical analysis provides properties of conflicting paths that have been exploited to devise improved mathematical models and solution algorithms for FQRP-G. In particular: ILP formulates the problem on a static graph model, whereas the formulations proposed by literature for problems related to FQRP-G rely on a dynamic (time-expanded) graph, thus requiring a larger number of variables and constraints; Heuristic A fairly improves the computational complexity with respect to the implementation proposed by [7];
- ILP, by means of state-of-the-art off-the-shelf mathematical optimization software, can solve instances of up to 100 vehicles in a few seconds at most, providing the minimum number of horizontal lanes and related routes. ILP can even solve larger instances of up to hundreds of vehicles, at the cost of longer running times, which may be not compliant with real route-execution environments;
- one of the proposed heuristics, called Heuristic A, is very efficient and effective, even for instances with hundreds of vehicles. It always runs in negligible time, and, with only rare exceptions that never showed up in random benchmarks, it finds routing paths requiring just a few horizontal lanes (one or two) more than the optimal solution;
- from a routing network design perspective, our empirical study shows that a grid with four or five horizontal lanes normally allows for finding collision-free nonstop Manhattan paths for all the vehicles of FQRP-G. With such sizing, the needing to integrate the routing system with further state-of-the-art algorithms for multi-agent pathfinding (as to optimize possible delays and deviations from shortest routes) is rare and limited to some infrequent exceptions where the methods proposed in this paper would require higher grids.

Further research is needed towards heuristic algorithms that, like B1 or B2, do not rely on one-way lanes and on simple Manhattan paths, which, according to the theoretical results reviewed in this work, is mandatory to enable a smaller number of required levels for the instances that are critical for Heuristic A. Possible lines for future studies could also involve exact solution methods for FQRP-G, based on either the model proposed in this work or alternative mathematical programming formulations, and the extension of FQRP-G and related solution approach to more and more realistic settings, e.g., considering arbitrary vehicle origins and destinations or more general grids.

## Figures and Tables

**Table 1:** Experimental results of ILP and Heuristic A on random instances.

Instance	ILP			Heur A			
*n*	Avg	Min–Max	Time	Avg	Err%	Min–Max	$\Delta_{\max}$
10	2.2	2–3	0.02	2.25	0.0	2–3	0
25	2.4	2–3	0.09	2.95	15.0	2–4	1
50	3.0	3–3	0.79	3.50	20.0	3–5	1
75	2.9	2–3	1.75	3.45	15.0	3–4	1
100	3.1	3–4	5.90	3.75	23.3	3–5	2
150	3.0	3–3	42.54	4.05	30.0	3–5	2
200	3.0	3–3	151.33	4.25	40.0	3–5	2
300	3.0	3–3	1099.91	4.30	43.3	4–5	2

**Table 2:** Experimental results of Heuristics B1 and B2 on random instances.

	Heur B1						Heur B2					
*n*	Succ%	Avg	Err%	Min–Max	$\Delta_{\max}$	Win-Lose%	Succ%	Avg	Err%	Min–Max	$\Delta_{\max}$	Win-Lose%
10	90	2.72	50.00	2–4	2	5–40	90	2.72	50.00	2–4	2	5–40
25	40	3.75	56.25	2–5	3	5–30	55	3.64	55.56	2–5	3	10–35
50	5	4.00	33.33	4–4	1	0–0	5	3.00	0.00	3–3	0	100–0
≥75	0	–	–	–	–	–	0	–	–	–	–	–

**Table 3:** Experimental results of ILP and Heuristic A on ad hoc instances.

Instance		Heur A	ILP	
n	Longest C-Path	Used Levels	Used Levels	Time
105	27	28	4	2.88
117	30	31	4	2.63
129	33	34	4	4.11
141	36	37	3	4.44
153	39	40	4	10.50
161	41	42	4	7.49
173	44	45	4	10.63
189	48	49	4	16.92
201	51	52	4	13.61
221	56	57	4	22.11
233	59	60	4	38.05

## Data Availability

Data are available from the corresponding authors.
